# Disrupted Alternative Splicing of RAB11FIP3 Contributes to Diabetic Foot Ulcer Dysfunction

**DOI:** 10.1111/jcmm.70663

**Published:** 2025-08-04

**Authors:** Dong Zhu, Feifei Chen, Xiaoyue Li, Qianqian Ning, Jian Wang, Wuhan Wei, Jingyu Zhang, Caiqi Shen, Lili Sun, Jiawen Gao, Ziyi Wang, Yuting Liu, Aijun Zhang, Qiang Li, Peisheng Jin

**Affiliations:** ^1^ Department of Plastic Surgery Affiliated Hospital of Xuzhou Medical University Xuzhou China; ^2^ Xuzhou Medical University Xuzhou China; ^3^ Jiangsu Center for the Collaboration and Innovation of Cancer Biotherapy Cancer Institute, Xuzhou Medical University Xuzhou Jiangsu China

**Keywords:** alternative splicing (AS), DFUs, HIF‐1α, RAB11FIP3, ubiquitination

## Abstract

Dysregulation of alternative splicing (AS) has been associated with various complications of diabetes, yet its role in the pathogenesis of diabetic foot ulcers (DFUs) and its involvement in metabolic memory (MM) remain unclear. In this study, we identified specific AS events in RAB11FIP3, notably the full‐length (RAB11FIP3‐FL) isoform and exon 6 exclusion (RAB11FIP3‐Δ6). We found that RAB11FIP3 (FL/Δ6) ratio is significantly elevated in patients with MM and DFUs. Additionally, we demonstrated that knockdown of RAB11FIP3‐FL alleviates vascular endothelial damage associated with MM and enhances DFU healing. Furthermore, we identified that HNRNPL promotes the retention of exon 6 in RAB11FIP3‐FL, thereby increasing the FL/Δ6 ratio. Mechanistically, our results show that RAB11FIP3‐FL promotes the ubiquitination and degradation of HIF‐1α through NEDD4L, independent of VHL. In conclusion, our study identifies RAB11FIP3‐FL as a pathogenic splicing isoform contributing to impaired DFU healing. Knockdown of RAB11FIP3‐FL promotes vascular regeneration and accelerates diabetic wound healing, offering new therapeutic targets for DFU treatment.

**Abbreviations**
A3: alternative 3’ Splice Site
A5: alternative 5’ Splice Site
AF: alternative first exon
AL: alternative last exon
AS: alternative splicing
ASEs: alternative splicing events
ASO: antisense oligonucleotides
CHX: cycloheximide
co‐IP: co‐immunoprecipitation
DEAS: differentially expressed alternative splicing
DFUs: diabetic wound ulcers
ES: exon skipping
HIF‐1α: hypoxia‐inducible factor‐1α
HNRNPL: heterogeneous nuclear ribonucleoprotein L
HUVECs: human umbilical vein endothelial cells
MM: metabolic memory
MX: mutually exclusive exon
PSI: percent spliced in
RAB11FIP3‐FL: full‐length RAB11FIP3
RAB11FIP3‐Δ6: exon 6 exclusion RAB11FIP3
RI: retained intron
VHL: von Hippel—Lindau

## 1 Introduction

Diabetes is a chronic disorder characterised by dysregulated glycaemic conditions [1]. According to the World Health Organisation and the International Diabetes Federation, the global prevalence of diabetes has surged from 108 million cases in 1980 to 537 million in 2021, with projections estimating 700 million cases by 2045 [2]. Diabetic foot ulcers (DFUs) are among the most severe complications of diabetes, affecting 15%–25% of diabetic patients. Infections occur in approximately 50%–60% of foot ulcers, with 20% of moderate to severe cases resulting in lower limb amputation [3]. The five‐year mortality rate for patients with DFUs is around 30%, while for those undergoing amputation, it exceeds 70%. Chronic diabetic wounds pose a significant health and economic burden worldwide [4].

Metabolic memory (MM) refers to the persistent molecular modifications that develop in cells following transient hyperglycaemic exposure, which endure despite subsequent glycaemic normalisation. These alterations persist and continue to influence cellular function and behaviour, leading to a sustained impairment of wound healing [5, 6, 7]. MM is closely associated with an increased risk of several diabetes‐related complications, including cardiovascular disease, retinopathy, kidney disease and DFUs [8, 9, 10, 11]. Notably, studies have revealed that metabolic memory induces long‐term adverse effects on microvascular and macrovascular systems, with antidiabetic drugs and antioxidants shown experimentally to be insufficient in fully reversing these impacts [12, 13, 14]. This underscores the critical need to deepen our understanding of metabolic memory mechanisms to develop targeted interventions that can mitigate its enduring vascular damage and improve long‐term outcomes for diabetic patients.

Alternative splicing (AS) plays a major role in enhancing the complexity of transcriptomes in multicellular eukaryotic organisms [15]. The majority of multi‐exon human genes undergo AS, resulting in a single gene producing multiple distinct mature mRNAs, thus expanding the cell's protein‐coding repertoire [16, 17]. While the most common disease associated with AS disorders in multiple genes is cancer [18, 19], many studies have identified disease‐causing splice variants in a wide range of diseases, from neuromuscular disorders to diabetes or cardiomyopathy [20]. However, the mechanism of AS in wound healing of DFUs and MM still needs further exploration.

In this study, we found that the RAB11FIP3 (FL/∆ 6) ratio is highly expressed in hyperglycaemia, MM and DFU patients. We identified the splicing factor HNRNPL as an upstream regulator of RAB11FIP3. Our study indicated that the imbalance in the RAB11FIP3 (FL/∆6) ratio might contribute to sustained damage to vascular endothelial cells. Our findings suggest that RAB11FIP3‐FL promotes HIF‐1α ubiquitination and degradation via NEDD4L, a process that remains active even in the absence of VHL. Our findings highlight RAB11FIP3‐FL as a potential therapeutic target for improving DFU healing.

## 2 Methods and Materials

### 2.1 Cell Culture and Treatment

Human umbilical vein endothelial cells (HUVECs) were purchased from Cell Bank of the Chinese Academy of Sciences (Shanghai, China). Before the experiment, HUVECs were cultured in DMEM (KeyGen Biotech, Jiangsu, China) containing 10% foetal bovine serum (Clark Bioscience, Houston, USA) and were cultured at 37°C at 5% carbon dioxide in an incubator with humidity. Previous results showed that we employed 25 mM glucose concentration as an in vitro experiment to simulate a high glucose environment [21]. Cells at passages 3–5 were incubated in a medium containing either 25 mM high glucose (HG) or 5 mM normal glucose (NG) along with 25 mM mannitol as an osmotic control. To mimic the transient hyperglycaemia conditions in vitro, HUVECs were initially incubated in high glucose (HG) for 24 h and then cultured in normal glucose for 2 d (MM) [11].

All plasmids were purchased from GenePharma Technology (Shanghai, China). According to the instructions on the product side, Lipofectamine 2000 (Life Technologies, Carlsbad, CA, USA) was used for plasmid transfection.

### 2.2 Tissue Samples

The human tissue study was approved by the Ethics Committee of the Affiliated Hospital of Xuzhou Medical University and was carried out following the Declaration of Helsinki. Twelve samples were collected from diabetic wounds in the amputation group, while the twelve samples in the normal group were taken from patients undergoing debridement with normal blood glucose levels. All samples were sourced from the Affiliated Hospital of Xuzhou Medical University from 2022 to 2024[XYFY2020‐KL041‐01].

### 2.3 RT‐PCR and RT‐qPCR

Cell samples were lysed with TRIzol reagent (Invitrogen). Tissue samples were ground into fine powder in liquid nitrogen and lysed using TRIzol reagent (Invitrogen). Total RNA extraction followed the manufacturer's protocol. RNA was reverse transcribed into cDNA using a reverse transcription polymerase chain reaction kit (Vazyme). qRT‐PCR using SYBR Green (Vazyme) was performed on a LightCycler 480 instrument (Roche). The mRNA levels of human genes or mouse genes were assessed by qRT‐PCR. Each qRT‐PCR reaction was independently repeated at least three times to ensure reproducibility. The data were analysed by the 2^−ΔΔCT^ method. The primers for RT‐qPCR and RT‐PCR were designed by an online tool (Primer3web) as indicated in Table S2.

### 2.4 Western Blot Analysis

In simple terms, cell extracts were separated on 4%–12% prefabricated gel SDS‐PAGE, and then the proteins were transferred to nitrocellulose membranes and incubated overnight at 4°C with primary antibody (1:2000 dilution) followed by secondary antibody (1:4000 dilution) at room temperature for 1.5 h. Immune response bands were identified using Tanon Scanning equipment (Tanon Science & Technology). Details about the antibodies used can be found in Table S1.

### 2.5 Cell Migration, Wound Healing and Tube Formation Assays

For migration assay, the migration ability of HUVECs was also tested by transwell chamber (8 μm pores, LABSELECT, China). Adjust the cell concentration to 2.5 × 10^5^ cells/ml with serum‐free medium, and then 200 μL cell suspension was added to the upper chamber of the migration well. To the contrary, 600 μL of complete medium was added to the lower chamber. 24 h later, cells in the upper compartment of transwell chambers were gently wiped with a cotton swab, then the migrated cells in the lower chamber were fixed with 4% paraformaldehyde for 30 min and finally stained with crystal violet for 20 min. The migrated or invaded cells were quantified by counting in five random fields under a microscope (Olympus, Center Valley, USA). Each experiment was analysed in triplicates. Wound healing and tube formation assays were performed as previously describe [21].

### 2.6 EdU

EdU assays were conducted to assess cell proliferation according to the protocol provided by the Cell‐Light EdU Apollo567 In Vitro Kit (RiboBio, Guangzhou, China). Cells were plated in 24‐well plates, cultured for 24 h and then followed by a 2 h incubation with 10 μM EdU solution in a cell incubator. Afterward, the cells were fixed in 4% paraformaldehyde for 30 min, permeabilized with 0.3% Triton for 10 min. The cells were then stained with the Apollo detection mixture for 30 min. Finally, the cells were counterstained with Hoechst 33,342 for 10 min, and representative images were obtained using a Olympus microscope (Center Valley, USA). The cell proliferation rate was calculated using the proportion of EdU‐positive cells (red) to Hoechst‐positive cells (blue). The manufacturers and catalogue numbers of all kits and materials are in Table S4.

### 2.7 Coimmunoprecipitation

To assess protein interactions, cells were lysed in 500 μL coimmunoprecipitation buffer, centrifuged at 12,000 g for 30 min, and underwent coimmunoprecipitation with magnetic beads and specific antibodies. The complex was washed overnight at 4°C with coimmunoprecipitation buffer to remove non‐specific bindings. Finally, the samples were mixed with SDS loading buffer and analysed using Western blotting to visualise the protein interactions.

### 2.8 Alternative Splicing Event Analysis

We utilised SUPPA2 v2.3 [22] to calculate seven types of alternative splicing events (ASEs) including A3/A5 (alternative 3′ and 5′ splice sites), AF/AL (alternative first and last exons), RI (retained intron), SE (skipping exon) and MX (mutually exclusive exon) by using the RNA‐seq annotations. Specifically, we executed the generateEvent command in SUPPA2, applied with the i options on the GTF file obtained from the GENCODE database. Subsequently, we calculated the PSI (Percent spliced in) levels for each ASE using the psiPerEvent command, based on the transcript‐level expression matrix produced by StringTie and the ioe file generated by the generateEvent step. The ASEs were further filtered to generate high‐confidence events by retaining events with an average PSI value ≥ 0.05 in ≥ 75% of samples. For detecting differentially spliced events, student's *t* test was used to detect differentially spliced events. Alternative splicing events with *p* value ≤ 0.05 and | ΔPSI| ≥ 0.1 were regarded as statistically significant in terms of differentially expressed alternative splicing (DEAS).

### 2.9 Establishment of Wound Model in Diabetic Nude Mice

Five‐week‐old male nude mice were sourced from GemPharmatech (Nanjing, China) and housed under aseptic conditions. All animal studies were authorised by the Animal Care and Ethics Committee of Xuzhou Medical University (Project number 202311 T024).

After 12 h of fasting, subjects' mice were induced with intraperitoneal streptozotocin (65 mg/kg; Sigma, Missouri, USA) dissolved in pH 4.5 citrate buffer. Then blood glucose was measured weekly, with mice exhibiting levels > 16.7 mmol/L for at least four weeks classified as diabetic. Animals were anaesthetised with Pentobarbital sodium; a full‐layer skin wound (diameter = 1.0 cm) was created on the mice's backs.

### 2.10 Assessment of Wound Closure

Thirty‐two diabetic nude mice and 8 normal nude mice were divided into five groups of eight. The groups included a blank group (normal wound healing), control group (PBS injected into diabetic mice), ASO‐NC group (ASO‐NC was injected), ASO‐RAB11FIP3‐FL group (ASO‐RAB11FIP3‐FL was injected) and ASO‐RAB11FIP3‐Δ6 group (ASO‐RAB11FIP3‐Δ6 was injected). Starting from the second day after wound establishment, ASO treatments were administered via local perilesional injection at 20 mg/kg per mouse, once every two days. The control group received PBS via the same route and frequency as ASO treatments. Wounds were photographed and analysed using Image J software on days 0 and 14 post‐wounding. For data measurement, three mice were randomly chosen. The wound healing rate is measured as follows: wound healing index (%) = (1‐unhealed wound area/original wound area) × 100%.

### 2.11 H&E and Masson Staining

The tissue sections are dewaxed and then soaked in distilled water. Next, they are stained with haematoxylin for 3–5 min and rinsed, followed by dehydration in 85% and 95% alcohol for 5 min each. Afterward, they immersed the sections in an eosin staining solution for 5 min. Finally, the dehydrated sections were treated with a Masson staining solution, washed with running water and observed under a microscope.

### 2.12 Statistical Analysis

Statistical analysis was conducted using SPSS 22.0, while GraphPad Prism 9 was used for data visualisation. Results are expressed as the mean ± standard deviation (SD). Statistical differences were assessed using Student's *t* test or one‐way analysis of variance (ANOVA), with a significance level set at *p* < 0.05.

## 3 Results

### 3.1 Identification of AS Events Caused by Transient Hyperglycaemia

**FIGURE 1:**
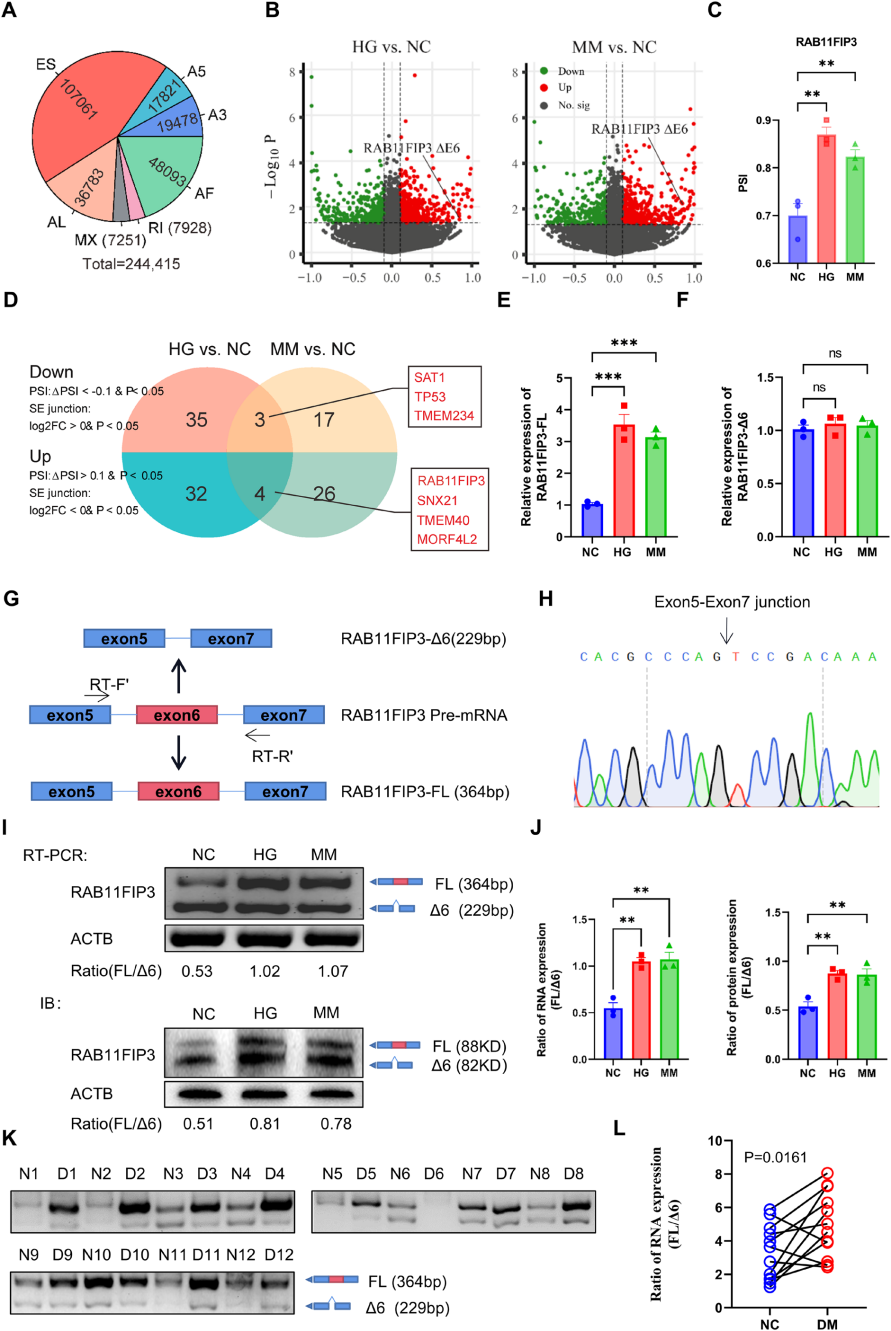
Identification of AS events in hyperglycemia. (A) Percentage of seven different splicing events. A5: Alternative 5’ Splice Site, A3: Alternative 3’ Splice Site, AF: Alternative First Exon, AL: Alternative Last Exon, MX: Mutually Exclusive Exon, RI: Retained Intron, and ES: Exon Skipping. (B) The volcano plot visualises the DEAS events between NC and HG, HG and MM. Red and green points in the plot represent statistically significant DEAS events. (C) Distribution of PSI value of RAB11FIP3 exon 6 skip events in HUVECs in NC, HG and MM. *p* values were calculated by two‐sided Mann–Whitney test, **p < 0.001. (D) Venn diagram resolution of differentially expressed genes under NC and HG, HG and MM conditions. (E,F) Differences of RAB11FIP3‐FL and RAB11FIP3‐Δ6 in under NC, HG and MM conditions. *p* values were calculated by two‐sided Mann–Whitney test, ***p < 0.001. ns, not significant. (G) The Scheme describes the primer design strategy to characterise the exon 6 inclusion or exclusion of RAB11FIP3. (F) Sanger sequencing analysis indicated the exon5‐exon7 junction in HUVECs. (H) RT‐PCR results of RAB11FIP3 in NC, HG, and MM. (I,J) Western blot and RT‐PCR analysis of RAB11FIP3 exon 6 skipping in HUVECs treated with NC, HG and MM conditions. (J) Quantitative analysis of the western blot and RT‐PCR results by the ImageJ software. Data were shown as mean ± SD. *p* values were calculated by two‐sided Student's *t* test, **p < 0.01. (K,L) RT‐PCR analysis of different RAB11FIP3 transcripts in normal subjects and DFU patients. (L)The statistical diagram of K (*N* = 12; Student *t*’ test).

For in vitro metabolic memory conditions caused by hyperglycaemia, we incubated HUVECs in high‐glucose (HG) medium for 24 h and then on normal glucose medium for 2 d (MM). We found that high glucose caused damage to HUVECs, which could not be reversed even after normal glucose was restored, which demonstrated that transient high glucose caused persistent damage to the vascular endothelial cells, leading to a decrease in the ability of vascular endothelial cells to proliferate, migrate and form tubes (Figure S1A–D). To explore the effects of MM on vascular endothelial cells, we identified a series of aberrant splice isoforms by RNA sequencing, with a total of 244,415 AS events, predominantly ES events (Figure 1A). A differential analysis of AS events was conducted between NC, HG and MM. As a result, 69,814 DEAS events with a *p* value < 0.05 and |∆PSI| > 0.1 (Figure 1B). Among the identified DEAS events, the PSI value of RAB11FIP3 (ENSG00000090565) and Ratio of RAB11FIP3(FL/∆6) was significantly elevated in HG and MM (Figure 1C and Figure S1J). Analysis based on PSI ratios revealed three genes that were upregulated in both HG and MM (SAT1, SAT1, TMEM234) and four genes that were downregulated in both MM and NC (TMEM40, MORF4L2, RAB11FIP3, SNX1) (Figure 1D). Next, we cultured HUVECs under MM and HG environments, and performed RT‐PCR experiments to validate the results, and finally identified RAB11FIP3‐FL and RAB11FIP3‐Δ6 (Figure 1I and Figure S1H). We designed 3 primer pairs, with primer set 1 covering a fragment spanning exon 5 to exon 7, to quantify the expression of RAB11FIP3‐FL and RAB11FIP3‐Δ6 transcripts (Figure 1G). In addition, primer 2 covering the exon 5/exon 6 and exon 6/exon 7 junctions was designed to validate the RAB11FIP3‐FL transcripts. Primer set 3 covering exon 5/exon 7 junctions was used to quantify RAB11FIP3‐Δ6 transcripts. Sanger sequencing results confirmed the presence of RAB11FIP3‐FL and RAB11FIP3‐Δ6 isoforms in HUVECs (Figure 1H). Next, the qRT‐PCR results indicated a significant increase in RAB11FIP3‐FL expression in HUVECs under HG and MM condition, while RAB11FIP3‐Δ6 levels remained unchanged (Figure 1E,F). Further analyses showed that HG remarkably enriched the ratio of RAB11FIP3(FL/∆6) at both RNA and protein level (Figure 1I,J). Similarly, clinical samples from DFUs showed an increased ratio of RAB11FIP3(FL/Δ6) compared to normal tissues (Figure 1K,L). The data suggest an imbalance in alternative splicing of RAB11FIP3 exon 6 in DFUs.

### 3.2 RAB11FIP3‐FL Inhibits Migration of Vascular Endothelial Cells

**FIGURE 2:**
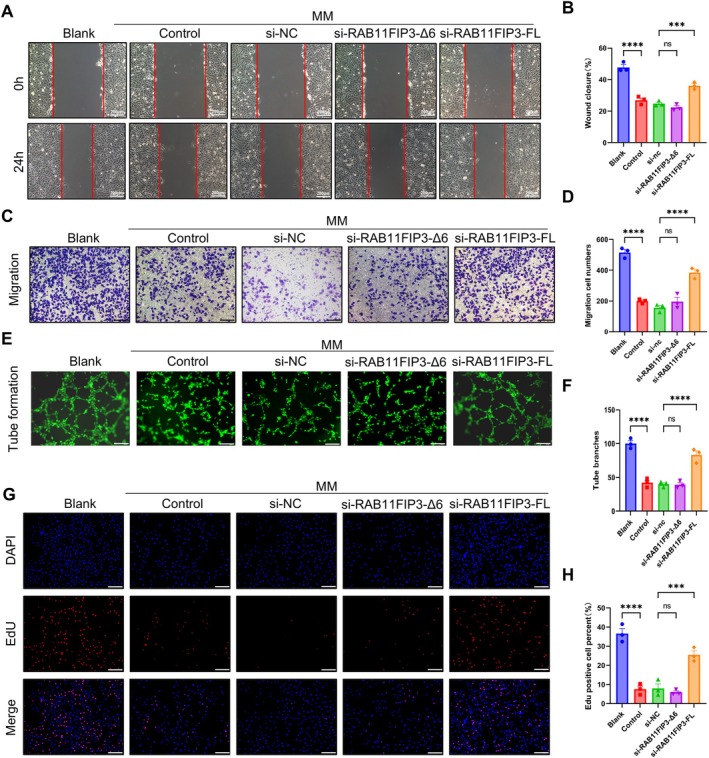
Knockdown of RAB11FIP3‐FL promotes the proliferation, migration, and tube‐forming ability of vascular endothelial cells in vitro. (A,B) Wound healing assay was performed to measure the motility of si‐RAB11FP3‐ Δ 6 and si‐RAB11FIP3‐FL in HUVECs under MM conditions. Quantification histogram represented wound closure rate (*n* = 3 biologically independent samples). Scale bar, 200 μm. (C,D) Representative images of transwell migration assay of HUVECs. Quantification histogram represented the number of migrated cells (*n* = 3 biologically independent samples). Scale bar, 200 μm. (E,F) Angiogenesis assay was performed to detect the metastatic ability of cells. Quantification histogram represented Tube branches (*n* = 3 biologically independent samples). (G,H) EdU incorporation assay was performed to assess DNA synthesis in HUVECs. Quantification histogram represented EdU positive cell percent (*n* = 3 biologically independent samples). Scale bar, 200 μm.

Considering that RAB11FIP3‐FL is highly expressed in both HG and MM environments and is also highly expressed in clinical samples, we conjecture that RAB11FIP3‐FL may be a pathogenic isoform of the MM phenomenon. To test our hypothesis, we then performed transwell and wound healing assays and found that si‐RAB11FIP3‐FL significantly promoted the ability of cell migration in HUVECs (Figure 2A–D). In addition, si‐RAB11FIP3‐FL significantly promoted the cell proliferation and tube‐forming ability of vascular endothelial cells (Figure 2E–H). Likewise, our results showed that overexpression of RAB11FIP3‐FL significantly inhibited the ability of cell proliferation, cell migration and tube‐forming ability of endothelial cells (Figure S2A–J). Intriguingly, the expression of RAB11FIP3‐Δ6 had no significant effect on cell proliferation, migration and tube formation (Figure 2A–H and Figure S2A–J). In conclusion, our experiments demonstrate that RAB11FIP3‐FL inhibits the proliferation, migration and tube formation of HUVECs under MM conditions.

### 3.3 Knockdown of HNRNPL Promotes the Exclusion of Exon 6 in the RAB11FIP3

**FIGURE 3:**
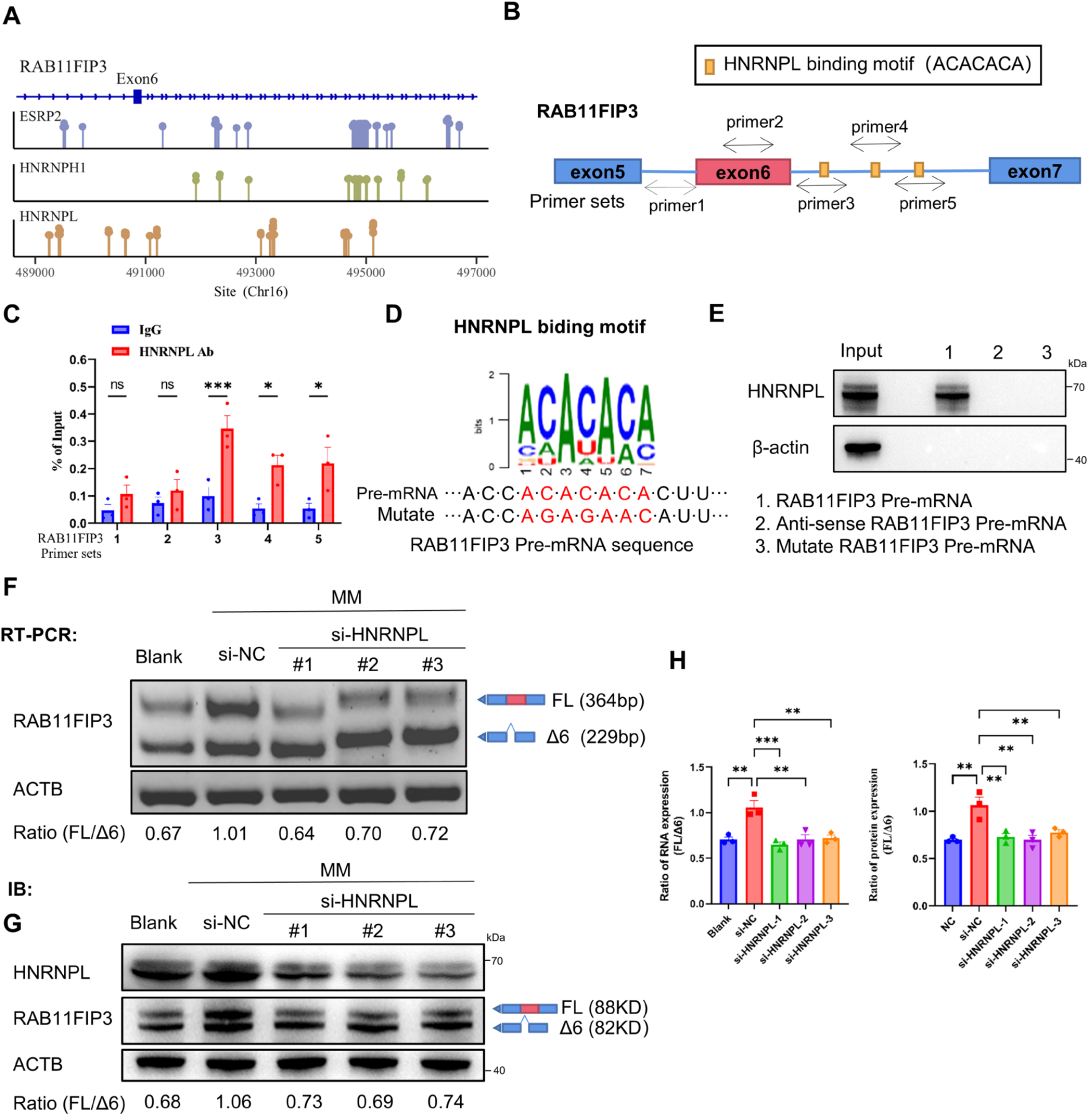
HNRNPL regulates AS events for RAB11FIP3. (A) This scheme describes the predicted splicing factors that may regulate RAB11FIP3. (B) A diagram of the human RAB11FIP3 pre‐mRNA with three HNRNPL‐binding motifs (yellow boxes) and the locations of the primer sets (colour‐coded arrows designated with the numbers 1–5 that were used for the RIP‐qPCR assays shown in (C). (C) The binding of HNRNPL to RAB11FIP3 pre‐mRNA was assessed by RIP‐qPCR using an anti‐HNRNPL antibody, followed by qPCR analysis with primer sets 1–5 (*n* = 3). IgG served as the control for the RIP‐qPCR assay. (D) Sequence of the HNRNPL binding motif (ACACACA) and its mutated version (AGAGAAC). (E) RNA Pull‐down assay showing that mutation of the ACACACA motif disrupted the binding between HNRNPL and RAB11FIP3 pre‐mRNA. (F) RT‐PCR analysis of RAB11FIP3 exon 6 skip after transfecting with si‐HNRNPL or control siRNA in HUVECs. (G) Western blot of RAB11FIP3 exon 6 skip after transfecting with si‐HNRNPL or control siRNA in HUVECs. (H) Quantitative analysis of the western blot and RT‐PCR results in F by the ImageJ software. Data were shown as mean ± SD. *p < 0.05, **p < 0.01 and ***p < 0.001.

Splicing factors play an important role in regulating the occurrence of AS events [23]. To gain deeper insights into how splicing factors regulate RAB11FIP3 exon 6 inclusion or exclusion, we utilised RBPmap (http://rbpmap.technion.ac.il/) to identify potential binding partners of RAB11FIP3 pre‐mRNA. The result identified three candidate splicing factors (ESRP, HNRNPH1, HNRNPL) potentially regulating exon 6 splicing (Figure 3A). Then, we individually silenced these splicing factor in HUVECs and performed RT‐PCR to assess the RAB11FIP3(FL/∆6) ratio (Figure S3A–C). Our results showed that the knockdown of HNRNPL significantly enhanced the exclusion of exon 6 in RAB11FIP3, while knockdown of ESRP or HNRNPH1 had no significant effect on its expression (Figure S3D–I). Further analysis confirmed that HNRNPL has multiple potential binding sites on RAB11FIP3 pre‐mRNA (Figure S3J). To validate this, we designed 5 primers targeting the enriched ACACACA motif near exon‐intron boundaries of exon 6 (Figure 3B). RIP‐qPCR confirmed HNRNPL binding to RAB11FIP3 pre‐mRNA, with significant enrichment at primer set 3 compared to IgG control (Figure 3C). To further confirm the role of the ACACACA motif, we introduced a mutation (AGAGAAC) into RAB11FIP3 pre‐mRNA, which disrupted its binding to HNRNPL (Figure 3D,E). Further analyses showed that HNRNPL knockdown remarkably attenuated the RAB11FIP3‐FL expression at both RNA and protein level (Figure 3F,G). Together, these findings demonstrate that HNRNPL directly binds to RAB11FIP3 pre‐mRNA and HNRNPL knockdown facilitates the exclusion of exon 6, highlighting its key role in alternative splicing regulation.

### 3.4 HNRNPL Inhibits the Proliferation, Migration and Tube Formation of HUVECs In Vitro

**FIGURE 4:**
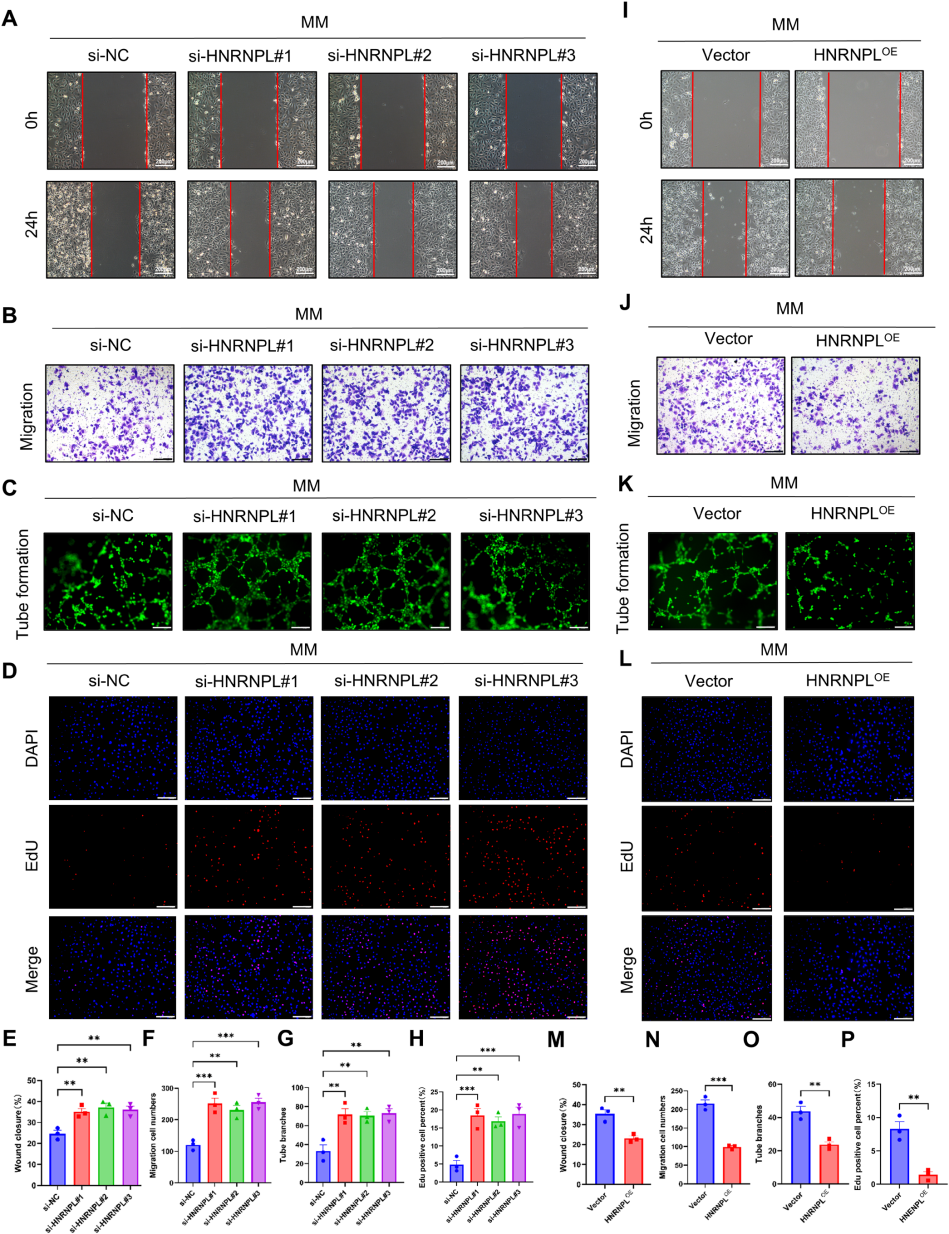
HNRNPL inhibits the proliferation, migration, and tube formation of HUVECs in vitro. (A) Wound healing assay was performed to measure the motility of si‐HNRNPL in HUVECs under MM conditions. Scale bar, 200 μm. (B) Representative images of transwell migration assay of si‐HNRNPL and HNRNPLOE in HUVECs under MM conditions. Scale bar, 200 μm. (C) Angiogenesis assay was performed to detect the metastatic ability of si‐HNRNPL in HUVECs under MM conditions. Scale bar, 200 μm. (D) EdU incorporation assay was performed to assess DNA synthesis of si‐HNRNPL in HUVECs. Scale bar, 200 μm. (I) Wound healing assay was performed to measure the motility of HNRNPLOE in HUVECs under MM conditions. Scale bar, 200 μm. (J) Representative images of transwell migration assay. Scale bar, 200 μm. (K) Representative images of tube formation assay. Scale bar, 200 μm. EdU incorporation assay. （L）The Edu incorporation assay was conducted to evaluate DNA synthesis in HUVEC with HNRNPL overexpression. (E,M) Quantification histogram represented wound closure rate (*n* = 3 biologically independent samples). (F,N) Quantification histogram represented the number of migrated cells (*n* = 3 biologically independent samples). (G,O) Quantification histogram represented Tube branches(*n* = 3 biologically independent samples). (H,P) Quantification histogram represented tube branches (*n* = 3 biologically independent samples). Each value is expressed as the mean ± SD of 3 independent experiments. *p < 0.05, **p < 0.01, ***p < 0.001, and ****p < 0.0001.

HNRNPL is a type of RNA‐binding protein predominantly located in the nucleus, and it is an important member of the HNRNP family [24]. Previous studies have shown that the expression of HNRNPL is increased in diabetes, and its high expression has negative effects on endothelial cells [25, 26]. Similarly, our research indicates that HNRNPL is highly expressed under hyperglycaemia metabolic memory conditions (Figure S3K). Subsequent experiments revealed that the knockdown of HNRNPL significantly enhanced cell proliferation, migration and tube formation of HUVECs under MM conditions (Figure 4A–H). Conversely, the overexpression of HNRNPL led to a notable inhibition of cell proliferation, migration and tube formation of HUVECs (Figure 4I–P). Moreover, intriguingly, the detrimental effects induced by HNRNPL overexpression could be mitigated by the knockdown of RAB11FIP3‐FL (Figure S4A–E). Collectively, these findings suggest that HNRNPL leads to endothelial injury by promoting the inclusion of exon 6 of RAB11FIP3 and the expression of RAB11FIP3‐FL. However, the precise impact of RAB11FIP3‐FL on the proliferation and migration abilities of endothelial cells remains uncertain.

### 3.5 RAB11FIP3‐FL Enhances Ubiquitination‐Mediated HIF‐1α Degradation

**FIGURE 5:**
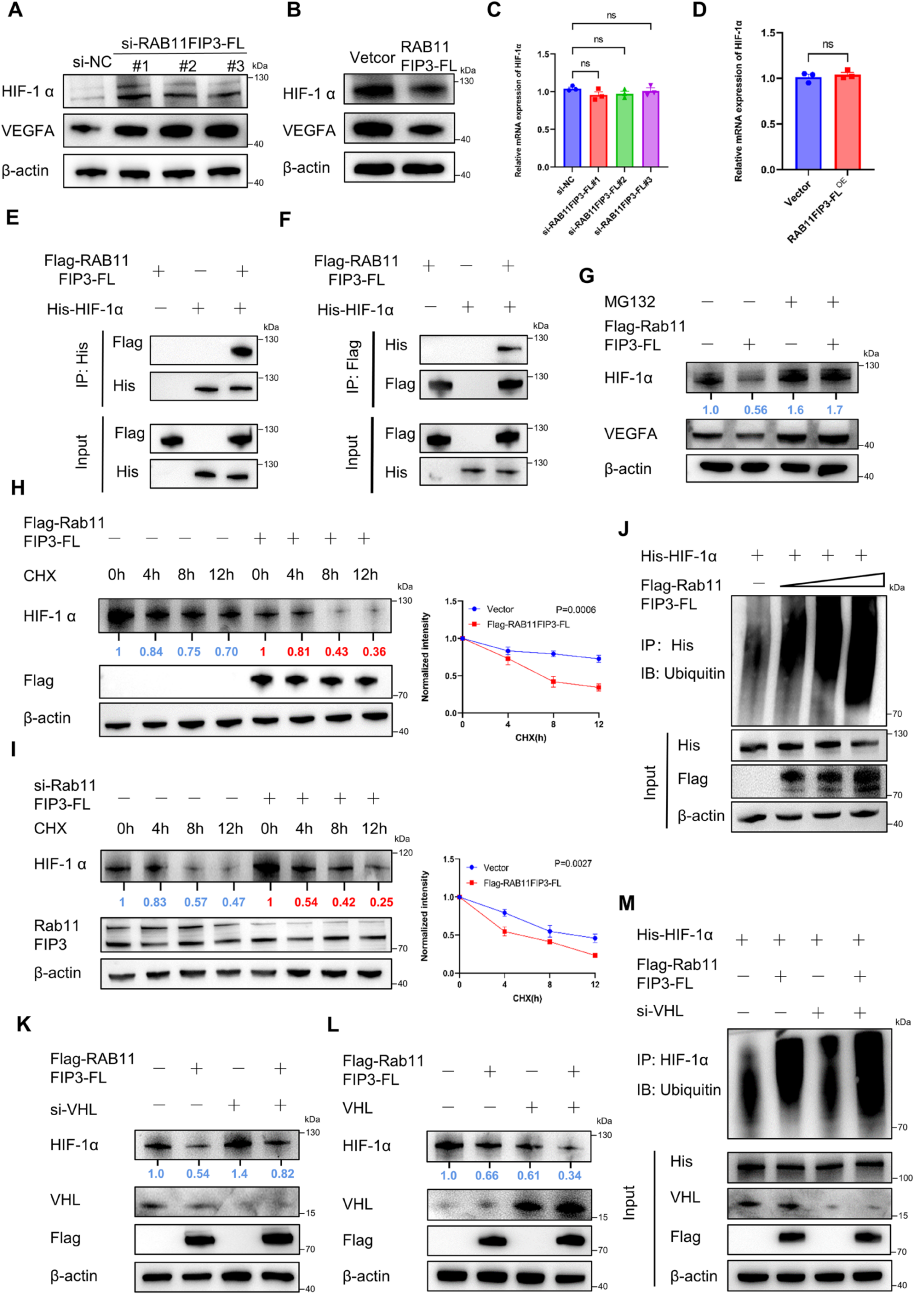
RAB11FIP3‐FL facilitates HIF‐1α ubiquitination and degradation even when VHL is depleted. (A) Immunoblot analysis showing HIF‐1α expression after HUVECs transfection with si‐RAB11FIP3‐FL. (B) Immunoblot analysis showing HIF‐1α expression after HUVECs transfection with the indicated RAB11FIP3‐FL overexpression plasmid. (C,D) The mRNA levels of HIF‐1α in HUVECs were measured using qRT‐PCR. (E,F) Flag‐RAB11FIP3‐FL and His‐HIF‐1α were co‐transfected into HUVECs. Co‐IP was performed to demonstrate the interactions between exogenous RAB11FIP3‐FL and HIF‐1α. (G) After treatment with MG132(20 mM) for 4 h, HIF‐1α protein expression in HUVECs was detected by immunoblot analysis. (H,I) Immunoblot analysis showing the turnover rate of HIF‐1α in HUVECs upon Flag‐RAB11FIP3‐FL overexpression or knockdown (left). Cells were treated with CHX (50 mg/mL) for the indicated time. The relative abundance of remaining HIF‐1α protein was normalised to ACTB and then normalised to baseline (*t* = 0) controls (right). (J) Immunoblot analysis of poly‐ubiquitinated HIF‐1α in poly‐ubiquitination assays of HUVECs transfected with the indicated constructs. (K) Immunoblot analysis of HIF‐1α level Co‐transfect HUVECs with RAB11FIP3‐FL and si‐VHL. (L) Co‐transfection of RAB11FIP3‐FL and VHL into HUVECs to analyse HIF‐1α expression by immunoblot analysis. (M) Co‐transfection of RAB11FIP3‐FL, His‐HIF‐1α and si‐VHL into HUVECs to analyse HIF‐1α expression by immunoblot analysis.

HIF‐1α and VEGF play pivotal roles in vascular regeneration [27]. We are interested in investigating whether RAB11FIP3‐FL influences the expression of HIF‐1α and VEGF. Our study revealed a decrease in the expression of HIF‐1α and VEGF after silencing RAB11FIP3‐FL (Figure 5A). Concurrently, the overexpression of RAB11FIP3‐FL significantly decreased HIF‐1α protein expression (Figure 5B). Interestingly, our data showed that the mRNA expression of HIF‐1α was not significantly changed regardless of whether RAB11FIP3‐FL was knocked down or overexpressed (Figure 5C,D). These results suggest that RAB11FIP3‐FL may inhibit angiogenesis by reducing the expression levels of HIF‐1α and VEGF. Exogenous co‐IP showed that HIF‐1α was co‐precipitated by RAB11FIP3‐FL, indicating the interaction between these two proteins (Figure 5E,F). Moreover, RAB11FIP3‐FL‐mediated HIF‐1α/VEGFA downregulation could be rescued by the proteasome inhibitor MG132 (Figure 5G), suggesting the involvement of proteasome/ubiquitin‐mediated degradation. RAB11FIP3‐FL increased the turnover rate of HIF‐1α, whereas the turnover rate of HIF‐1α was decreased in cells with RAB11FIP3‐FL on the basis of treatment with cycloheximide (CHX), which blocks protein synthesis (Figure 5H,I).

VHL‐dependent ubiquitin‐proteasomal degradation is the primary regulatory mechanism regulating HIF‐1α stability [28]. To determine whether RAB11FIP3‐FL affected ubiquitination and proteasomal degradation of HIF‐1α via VHL, we transfected Flag‐RAB11FIP3‐FL into HUVECs. Our results showed that RAB11FIP3‐FL facilitated the ubiquitination of HIF‐1α even in the absence of VHL (Figure 5M). Furthermore, RAB11FIP3‐FL suppressed steady‐state expression of HIF‐1α, regardless of VHL depletion (Figure 5K). Moreover, the overexpression of RAB11FIP‐FL was able to synergistically collaborate with VHL in facilitating the degradation of HIF‐1α (Figure 5L). In conclusion, all these data suggest that RAB11FIP3‐FL undermines the stability of HIF‐1α through ubiquitination and proteasomal degradation, and this process is independent of the role of VHL.

### 3.6 RAB11FIP3‐FL Associates With NEDD4L to Promote HIF‐1α Ubiquitination and Degradation

**FIGURE 6:**
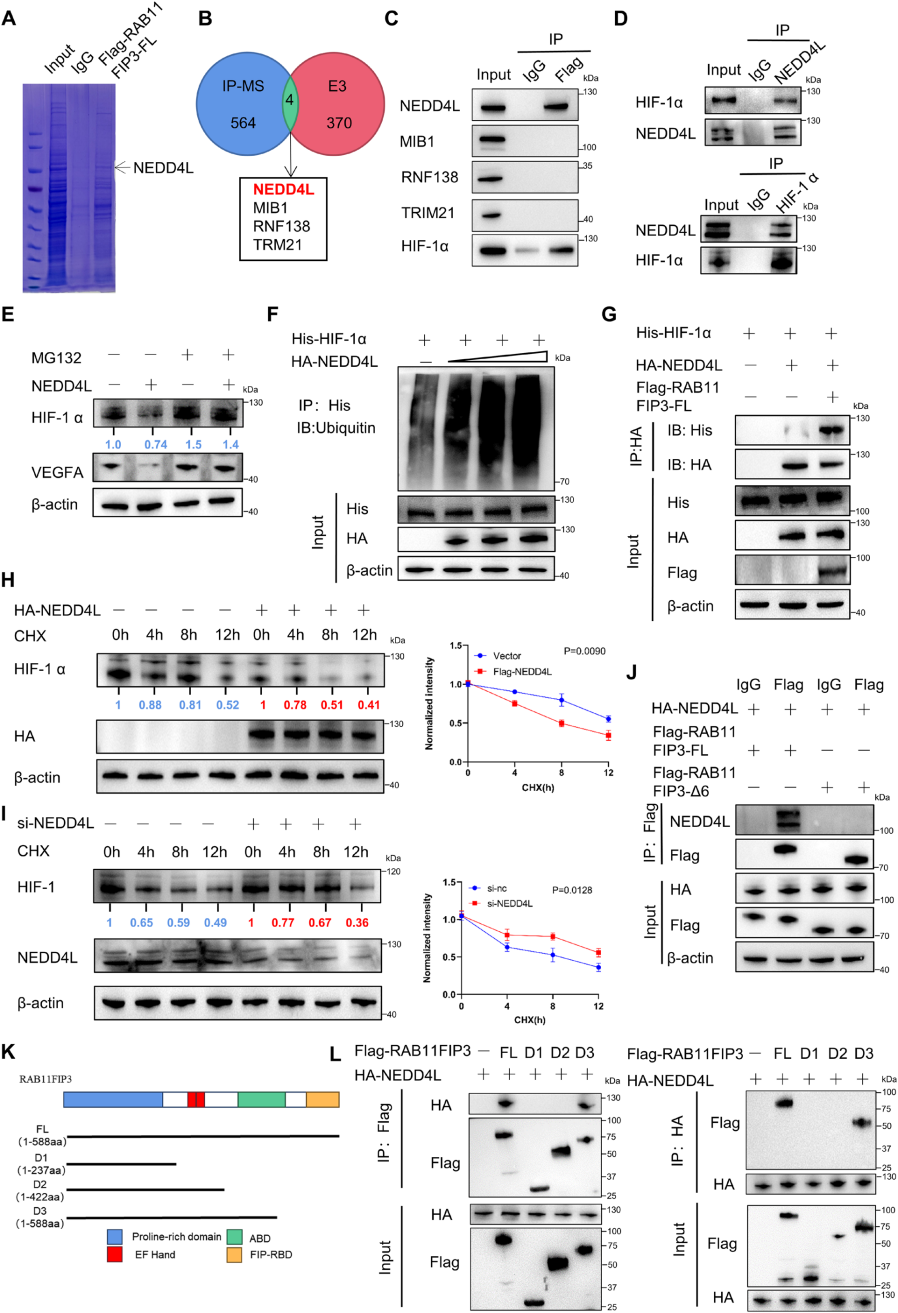
RAB11FIP3‐FL binds to NEDD4L and promotes HIF‐1α ubiquitination and degradation. (A) Co‐immunoprecipitation (Co‐IP) experiment visualised with Coomassie Brilliant Blue staining. (B) Venn diagram showing the overlap between co‐immunoprecipitated binding proteins and established human E3 ligase libraries identified using UbiNet 2.0. (C) The interactions between endogenous RAB11FIP3‐FL and NEDD4L, MIB1, RNF138, TRIM21 and HIF‐1α were analysed using a co‐IP assay. (D) Co‐IP assays demonstrated the interaction between HIF‐1α and NEDD4L in HUVECs. (E) Immunoblot analysis of HIF‐1α protein expression in HUVECs transfected with either the vector control or NEDD4L, following 4 h treatment with MG132 (20 mM). (F) Polyubiquitination assay showing polyubiquitinated HIF‐1α in HUVECs transfected with the indicated constructs, analysed by immunoblotting. (G) Co‐IP assays in HUVECs co‐transfected with His‐HIF‐1α, HA‐NEDD4L, and Flag‐RAB11FIP3‐FL, using anti‐HA and anti‐His antibodies. (H,I) Turnover rate of HIF‐1α in HUVECs upon HA‐NEDD4L overexpression or knockdown, determined by immunoblot analysis (left). Cells were treated with cycloheximide (CHX, 50 μg/mL) for the indicated times, and HIF‐1α levels were normalised to *β*‐actin and baseline controls (*t* = 0) (right). （J）Co‐IP assays were performed in HUVECs co‐transfected with HA‐NEDD4L, Flag‐RAB11FIP3‐FL and Flag‐RAB11FIP3‐Δ6 using anti‐Flag and anti‐NEDD4L antibodies. (K) Schematic diagram of full‐length RAB11FIP3 and its deletion mutants. (L) Co‐IP assays using HA‐NEDD4L and either full‐length RAB11FIP3‐FL or its deletion mutants, performed with anti‐HA or anti‐Flag antibodies.

Therefore, RAB11FIP3‐FL may promote HIF‐1α ubiquitination via an E3 ligase distinct from VHL. To identify the involved E3 ligase, we conducted mass spectrometry analysis (Figure 6A). By intersecting the 564 proteins identified through mass spectrometry with known E3 ligases, we identified four candidates: NEDD4L, MIB1, RNF138 and TRM21 (Figure 6B). Immunoprecipitation experiments revealed that only NEDD4L could interact with RAB11FIP3‐FL, and we also observed an interaction between RAB11FIP3‐FL and HIF‐1α (Figure 6C). Furthermore, co‐immunoprecipitation experiments confirmed the interaction between NEDD4L and HIF‐1α (Figure 6D and Figure S4G). These results suggest the formation of a ternary complex involving RAB11FIP3‐FL, NEDD4L and HIF‐1α.

To further validate this mechanism, we co‐transfected HA‐NEDD4L, FLAG‐RAB11FIP3‐FL and His‐HIF‐1α into HUVECs. The results showed that RAB11FIP3‐FL significantly enhanced HIF‐1α binding to NEDD4L, indicating its facilitative role (Figure 6G). Taken together, these findings demonstrate that RAB11FIP3‐FL recruits the E3 ligase NEDD4L to promote HIF‐1α ubiquitination and degradation, thereby providing a new regulatory mechanism for HIF‐1α turnover.

Next, we investigated whether NEDD4L affects HIF‐1α expression. Western blot analysis revealed that NEDD4L suppressed HIF‐1α expression, and this downregulation could be reversed by the proteasome inhibitor MG132 (Figure 6E). Additionally, NEDD4L promoted HIF‐1α degradation in a dose‐dependent manner (Figure 6F). Cycloheximide (CHX) chase assays further demonstrated that NEDD4L increased HIF‐1α turnover, whereas NEDD4L knockdown reduced HIF‐1α turnover (Figure 6H,I). These findings confirm that NEDD4L functions as a specific E3 ligase for HIF‐1α, providing critical insight into how HIF‐1α stability is regulated under diabetic conditions.

Notably, our immunoprecipitation results showed that NEDD4L interacts exclusively with RAB11FIP3‐FL, while failing to bind to RAB11FIP3‐Δ6 (Figure 6J). To further explore this interaction, we designed truncated constructs of RAB11FIP3. Domain localization experiments demonstrated that NEDD4L binds strongly to full‐length RAB11FIP3‐FL and the D3 truncated construct (amino acid residues 1–588), but not to the D1 (1–237aa) or D2 (1–422aa) constructs that lack the ABD domain (Figure 6E,F). This strongly suggests that aberrant splicing of exon 6, resulting in a defective ABD domain, is the primary cause of the disrupted interaction between RAB11FIP3‐Δ6 and NEDD4L.

### 3.7 ASO‐RAB11FIP3‐FL Promotes the Healing of Diabetes Wounds

**FIGURE 7:**
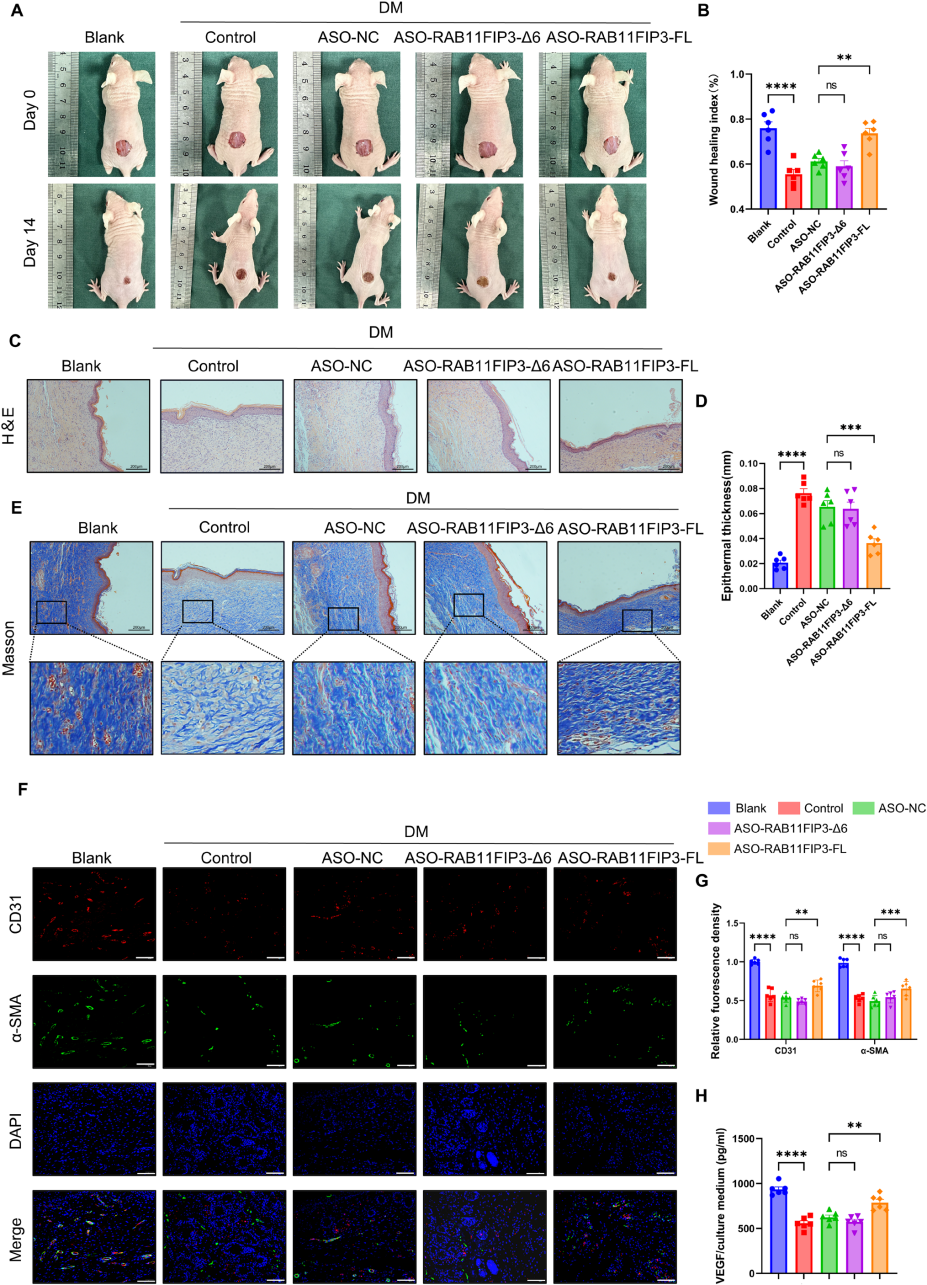
Knockdown of ASO‐RABl1FIP3‐FL promotes wound healing in diabetic mice. (A) Representative images of the wound area by different treatments on days 0 and 14 after operation. (B) Quantification histogram represented wound healing rate (*n* = 6 biologically independent samples). (C) H&E staining showed the regeneration of diabetic wounds. Bar = 200 μm. (D) Quantification histogram represented epithelial thickness (*n* = 6 biologically independent samples). (E) Representative images of Masson's trichrome stating of wound sections. Scale bar, 200 μm. (F,G) Immunofluorescence staining of α‐SMA and CD31‐positive cells. Bar =200 μm. (H) ELISA was used to detect VEGF during skin wound repair. Each value is expressed as the mean ± SD of 3 independent experiments. *p < 0.05, **p < 0.01, ***p < 0.001, and ****p < 0.0001.

Antisense oligonucleotides are widely used in clinical practice, including the treatment of diabetic wounds [29].we designed ASO targeting the degradation of RAB11FIP3‐FL and RAB11FIP3‐Δ6 (Table S3), and injected ASOs around the periphery of wounds to observe wound healing rates. Our study found that mice injected with ASO‐RAB11FIP3‐FL had a significantly faster wound healing rate, while the wound margin of mice with ASO‐RAB11FIP3‐Δ6 showed no significant change from the control group (Figure 7A,B). Re‐epithelialization is critical for wound healing. We found that ASO‐RAB11FIP3‐FL also improved re‐epithelialization as well as decreased the excessive inflammatory cell infiltration and collagen deposition (Figure 7C,D). Histological analysis of Masson's trichrome‐stained sections indicated that on day 14 post‐wounding, the collagen fibres in the ASO‐RAB11FIP3‐FL treated diabetic wounds were thicker and more regular compared with the control group (Figure 7E). Moreover, there was no statistically significant difference in ASO‐RAB11FIP3‐Δ6 compared to controls. As a marker of vascular endothelial cells, increased CD31 content reflects elevated vascular density [30], whereas α‐SMA serves as a marker for vascular smooth muscle cells, with upregulated expression indicating proliferation or activation of vascular wall smooth muscle cells [31]. Notably, immunofluorescence assays indicated that the expression levels of CD31 and α‐SMA were higher in the ASO‐RAB11FIP3‐FL group than in the control group (Figure 7F,G). In addition, we excised skins from mice, homogenised them and conducted an analysis of VEGF secretion within the wounds. Mice injected with ASO‐RAB11FIP3‐FL were found to stimulate the secretion of VEGF (Fig, 7H). In conclusion, our experiments indicate that the knockout of RAB11FIP3‐FL enhances the proliferation, migration and tube formation abilities of vascular endothelial cells. Furthermore, the knockout of RAB11FIP3‐FL promotes the expression of VEGF, which in turn facilitates the regeneration of blood vessels at the wound site and accelerates the healing process in diabetic wounds.

## 4 Discussion

**FIGURE 8:**
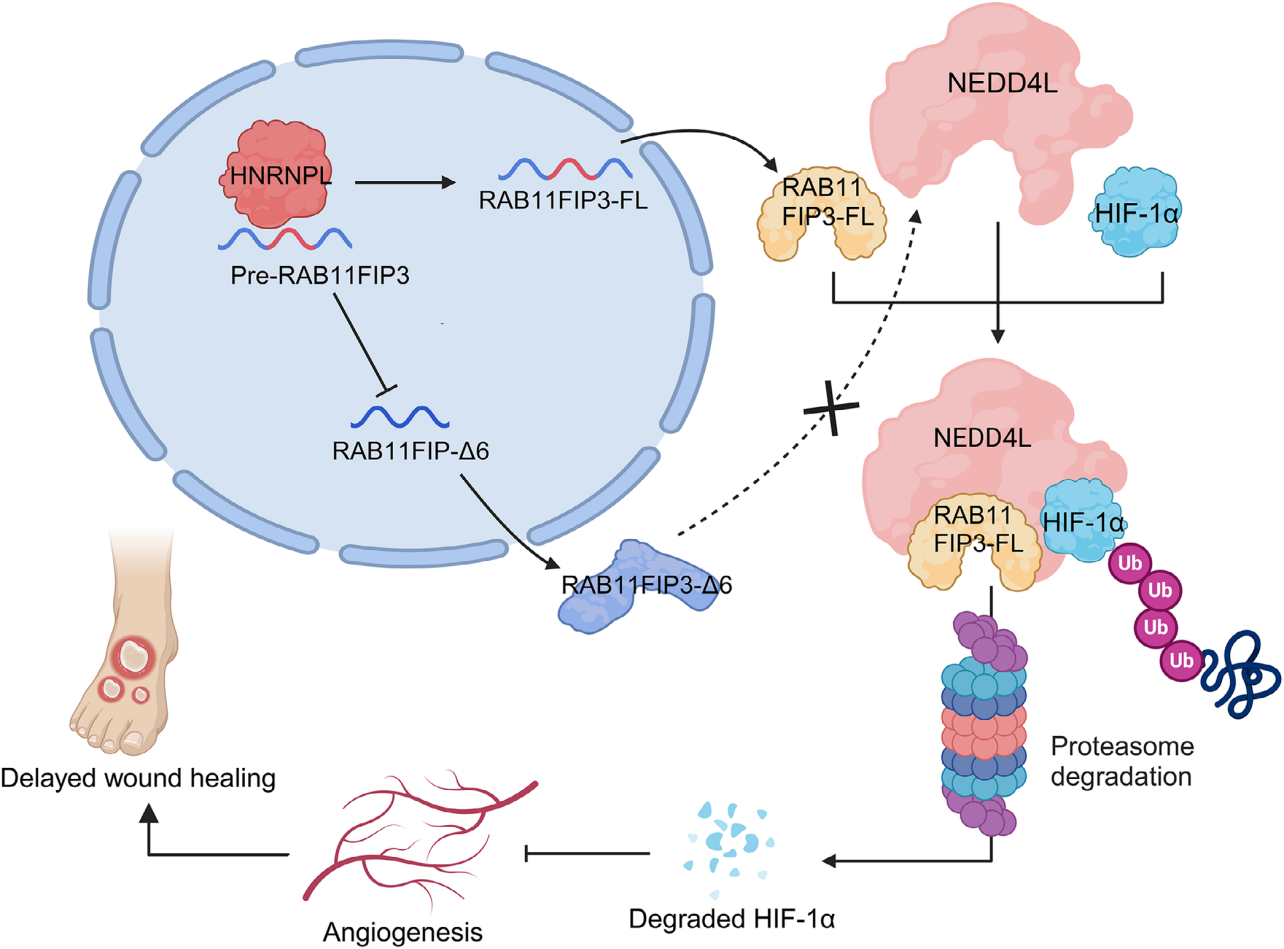
Schematic depiction of the mechanised wound healing in diabetic foot ulcers (DFUs).

In this study, we unveiled the elevated expression of RAB11FIP3‐FL under hyperglycaemic metabolic memory conditions and patients with DFUs, while no significant difference was observed in the expression of RAB11FIP3‐Δ6. Our findings suggest that the upregulation of RAB11FIP3‐FL may be associated with the persistent damage to vascular endothelial cells. HNRNPL promoted the retention of exon 6 in RAB11FIP3, thus regulating RAB11FIP3 (FL/Δ6). We characterised the E3 ubiquitin ligase NEDD4L that interacts with RAB11FIP3‐FL. Our results demonstrate that the RAB11FIP3‐FL/NEDD4L/HIF‐1α ternary complex facilitates the ubiquitin‐mediated degradation of HIF‐1α (Figure 8). This process subsequently impairs vascular regeneration in diabetic mice, leading to delayed wound healing.

AS serves as a pivotal mechanism in transcriptional regulation, generating transcript diversity and modulating protein structure and functionality [32]. Under physiological conditions, AS‐mediated protein diversity influences protein structure, function, interactions and localization, and thus participates in the differentiation and development of a range of tissues and organs. Under pathological conditions, AS mechanisms have been implicated in the pathogenesis of diabetes and its complications [20, 33]. RAB11FIP3 is involved in processes such as cell migration, cell cycle regulation and endosomal recycling [34, 35, 36], all of which may contribute to disease progression in DFU. Jing et al. demonstrated that RAB11FIP3 regulates the motility of breast cancer cells, but its specific role in DFUs requires further investigation [37]. We identified AS events in metabolic memory (MM) conditions and diabetic patients, revealing high expression of RAB11FIP3‐FL. In addition, our research indicates that targeting the knockdown of RAB11FIP3‐FL can promote the proliferation, migration, and tube formation ability of endothelial cells, and enhance wound healing in diabetic mice. Our research suggests that RAB11FIP3‐Δ6 does not have a significant effect on cell proliferation or migration.

HIF‐1 signalling pathways plays a crucial role in the healing process of diabetic wounds [38]. Studies suggest that hyperglycaemia leads to impaired HIF‐1 signalling pathways, weakening HIF‐1α mediated responses to hypoxia and resulting in the downregulation of its downstream target genes, ultimately leading to non‐healing diabetic foot ulcers [39, 40, 41]. Our research revealed that RAB11FIP3‐FL inhibits the expression of HIF‐1α in MM condition. Furthermore, RAB11FIP3‐FL regulates HIF‐1α through a post‐transcriptional manner by facilitating ubiquitin‐mediated proteasomal degradation of HIF‐1α, thus inhibiting its transcriptional activity. Admittedly, the oxygen‐dependent hydroxylation of HIF‐1α, mediated by VHL recruitment, has been well established as the primary pathway for HIF‐1α degradation [42, 43]. Our results show that RAB11FIP3‐FL can still mediate HIF‐1α ubiquitination in MM even when VHL is deleted, suggesting the involvement of other E3 ligases. Particularly, we identified the E3 ligase NEDD4L as a binding partner of RAB11FIP3‐FL and demonstrated that NEDD4L enhances the ubiquitin‐mediated degradation of HIF‐1α. Interestingly, we found that RAB11FIP3‐Δ6, a variant lacking exon 6, is unable to bind to NEDD4L. Exon 6 of RAB11FIP3 plays a crucial role in the formation of the ABD domain, which has been shown to be an important binding site for ARF proteins [35]. Our findings indicate that abnormal splicing of exon 6 leads to impaired synthesis of the ABD structural domain of RAB11FIP3, which may be responsible for preventing the binding of RAB11FIP3‐Δ6 to NEDD4L.

Alternative splicing of pre‐mRNA is mainly regulated by splicing factors. RNA‐binding proteins play a crucial role in the onset and progression of diabetes, as well as its systemic manifestations [44, 45]. HNRNPL mostly causes exon skipping, and this occurs when it is bound within cassette exons or to their flanking regions, owing to the presence of specific exon silencing sequences [46, 47]. We discovered that HNRNPL can promote the retention of exon 6 in RAB11FIP3‐FL, leading to an increased ratio of RAB11FIP3 (FL/Δ6). In in vitro experiments, we demonstrated that HNRNPL can inhibit the proliferation, migration and tube formation capability of endothelial cells, and this effect can be rescued by knocking down RAB11FIP3‐FL. This phenomenon further demonstrates that HNRNPL influences cellular functions under metabolic memory by regulating the expression of RAB11FIP3‐FL.

In conclusion, we explored a potential mechanism that the delayed healing of diabetic wounds was closely related to the upregulation of RAB11FIP3‐FL. HNRNPL may enhance the retention of exon 6 in RAB11FIP3‐FL, thereby increasing the ratio of RAB11FIP3 (FL/Δ6). RAB11FIP3‐FL can promote the ubiquitination and degradation of HIF‐1α through the combination of NEDD4L and HIF‐1 α, thus inhibiting the proliferation, migration and tubular formation of vascular endothelial cells and suppressing the healing of diabetes wounds. Our findings support the potential of RAB11FIP3‐FL as a promising treatment strategy for diabetic wound healing, with significant clinical application potential.

## Author Contributions

**Dong Zhu:** conceptualization (equal), investigation (lead), writing – original draft (lead). **Feifei Chen:** conceptualization (lead), data curation (equal), writing – original draft (equal). **Xiaoyue Li:** data curation (equal), investigation (equal), visualization (equal). **Qianqian Ning:** data curation (lead). **Jian Wang:** data curation (equal), investigation (equal), visualization (equal). **Wuhan Wei:** investigation (equal), visualization (equal). **Jingyu Zhang:** investigation (equal). **Caiqi Shen:** data curation (equal), funding acquisition (equal). **Lili Sun:** visualization (equal). **Jiawen Gao:** data curation (equal). **Ziyi Wang:** investigation (equal). **Yuting Liu:** investigation (equal). **Aijun Zhang:** visualization (equal). **Qiang Li:** conceptualization (equal), supervision (equal). **Peisheng Jin:** conceptualization (equal), funding acquisition (equal), supervision (lead).

## Ethics Statement

All samples were processed following the standard operating procedures with the appropriate approval of the Human Research Ethics Committees, including informed consent within the context of research (XYFY2020‐KL041‐01). Regarding animal studies, these were carried out only after securing authorisation from the Animal Care and Ethics Committee of Xuzhou Medical University (Project number: 202311 T024).

## Conflicts of Interest

The authors declare no conflicts of interest.

## Supporting information


Appendix S1.

## Data Availability

The data and the code that support the findings of this study are available at reasonable request from the corresponding authors.
