# Supplementary material for: Disrupted Alternative Splicing of RAB11FIP3 Contributes to Diabetic Foot Ulcer Dysfunction
Source: J Cell Mol Med. 2025 Aug 4;29(15):e70663. doi: 10.1111/jcmm.70663 (PMC12320866; doi:10.1111/jcmm.70663)
Supplement: Supplementary file 1 — Appendix S1. [file JCMM-29-e70663-s001.pdf]

Supplementary Figure1

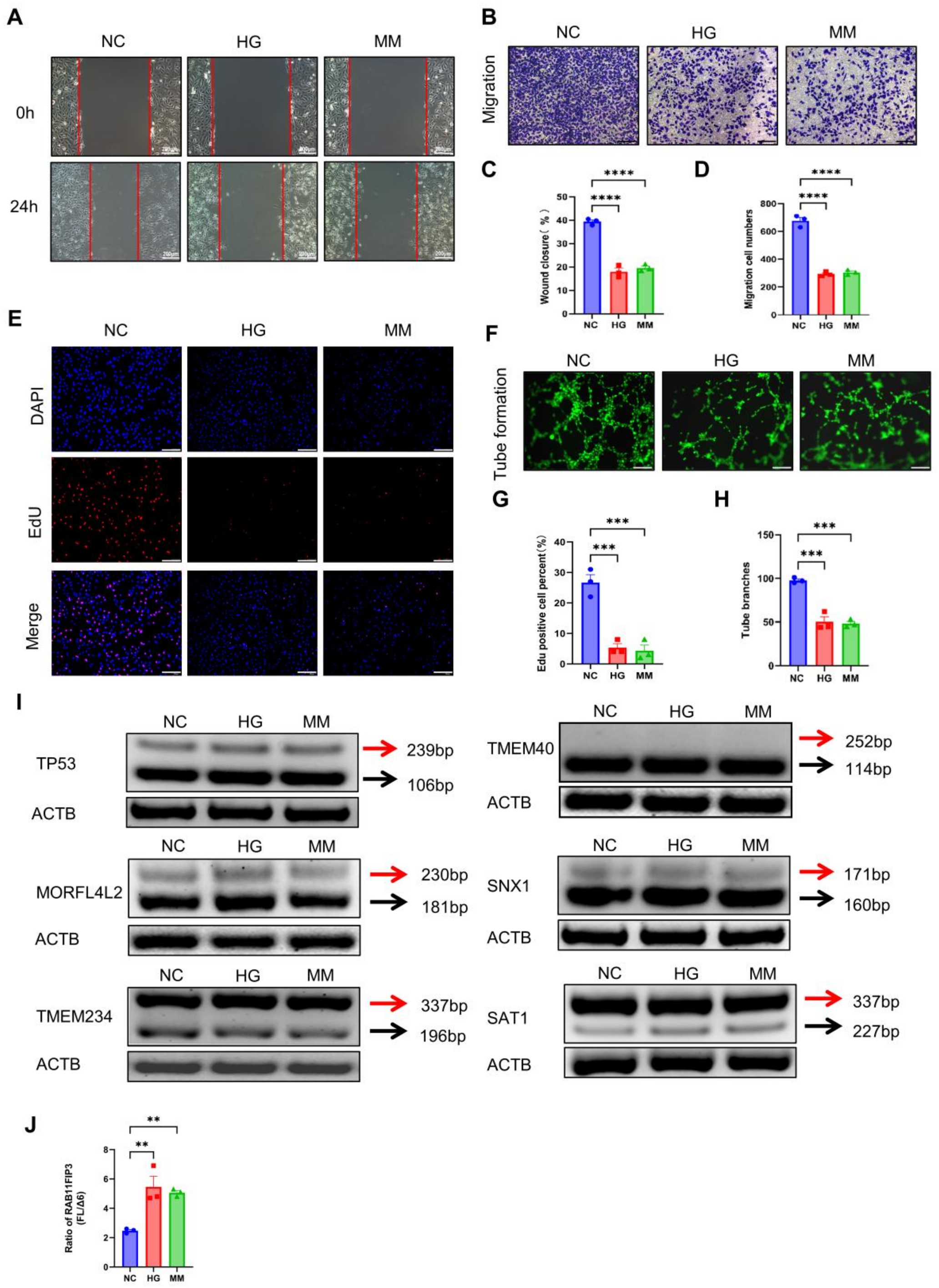

Supplementary Fig1. Effects of NC, HG, and MM conditions on HUVECs motility, migration, gene expression, and alternative splicing events. A Wound healing assay was performed to measure the motility of HUVECs under NC, HG, and MM groups. Scale bar, 200 µm. B Representative images of transwell migration assay of HUVECs under NC, HG, and MM groups. Scale bar, 200 µm. C

Quantification histogram represented wound closure rate. D Quantification histogram represented the number of migrated cells. E EdU incorporation assay was performed to assess DNA synthesis in HUVECs under NC, HG, and MM groups. Scale bar, 200μm. F Angiogenesis assay was performed to detect the metastatic ability of HUVECs under NC, HG, and MM groups. Scale bar, 200μm. G Quantification histogram represented EdU positive cell percent. H Quantification histogram represented Tube branches. I RT-PCR Analysis of Differential Transcript Expression of TP53, SAT1, TMEM234, TMEM40, MORF4L2, and SNX1 under NC, HG and MM conditions. J Differences of ratio of RAB11FIP3(FL/Δ6) in NC, HG and MM

Supplementary Figure2

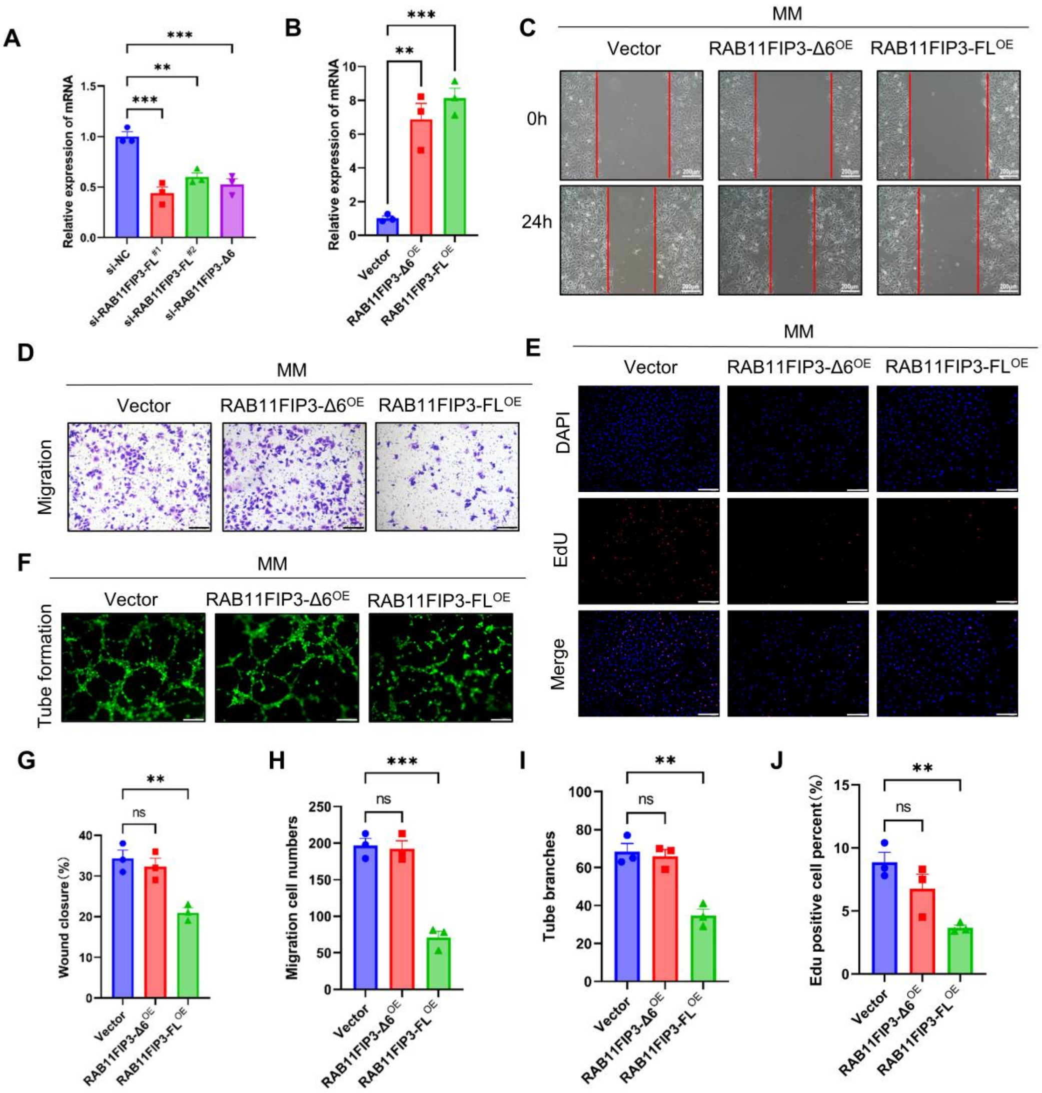

Supplementary Fig2. Characterization of RAB11FIP3-FL and RAB11FIP3-Δ6 expression and their effects on HUVECs motility, migration, angiogenesis, and proliferation. A Validate the knockout efficiency of RAB11FIP3-FL and RAB11FIP3- Δ 6 through q-PCR. B Verify the overexpression efficiency of RAB11FIP3-FL and RAB11FIP3- Δ 6 through q-PCR. C Wound healing assay was performed to measure the motility of HUVECs under the condition of overexpression of RAB11FIP3-FL and RAB11FIP3- Δ 6 respectively. D Representative images of transwell migration assay of HUVECs under the condition of overexpression of RAB11FIP3-FL and RAB11FIP3- Δ 6 respectively. E Angiogenesis assay was performed to detect the metastatic ability of HUVECs under the condition of overexpression of RAB11FIP3-FL and RAB11FIP3- Δ 6 respectively. F EdU incorporation assay was performed to assess DNA synthesis in HUVECs under the condition of overexpression of RAB11FIP3-FL and RAB11FIP3- Δ 6 respectively. G Quantification histogram represented wound closure rate. H Quantification histogram represented the number of migrated cells. I Quantification histogram represented Tube branches. J Quantification histogram represented EdU positive cell percent.

Supplementary Figure3

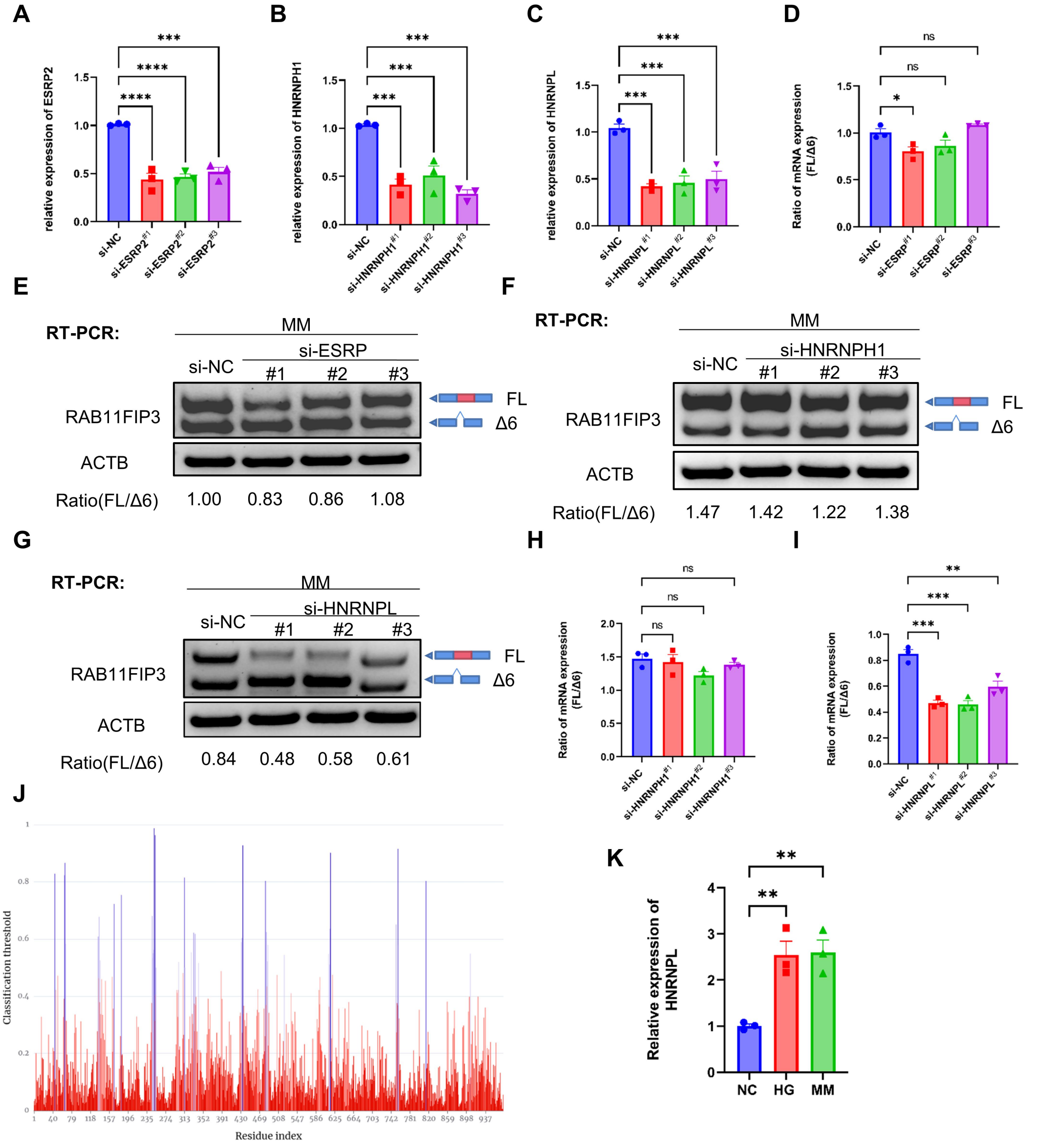

Supplementary Fig3. Analysis of ESRP2, HNRNPH1, and HNRNPL knockdown effects on RAB11FIP3 exon 6 splicing and differential expression in HUVECs. A qPCR analysis of ESRP2 mRNA in negative control (si-NC) and three sets of small interfering RNA for ESRP2 (si-ESRP2#1-3). B qPCR analysis of HNRNPH1 mRNA in negative control (si-NC) and three sets of small interfering RNA for HNRNPH1 (si-HNRNPH1#1-3). C qPCR analysis of HNRNPL mRNA in negative control (si-NC) and three sets of small interfering RNA for HNRNPL (si-HNRNPL$^{\#1\text{-}3}$). D Quantitative analysis of the RT-PCR results in E by the ImageJ software. E RT-PCR analysis of RAB11FIP3 exon 6

skip after transfecting with si-ESRP2 or control siRNA in HUVECs. F RT-PCR analysis of RAB11FIP3 exon 6 skip after transfecting with si-HNRNPH1 or control siRNA in HUVECs. G RT-PCR analysis of RAB11FIP3 exon 6 skip after transfecting with si-HNRNPL or control siRNA in HUVECs. H Quantitative analysis of the RT-PCR results in E by the ImageJ software. I Quantitative analysis of the RT-PCR results by the ImageJ software. J This predicts the binding sites between RAB11FIP3 and HNRNPL proteins. (Using the online tool PRIDictor (http://www.rnainter.org/PRIdictor/) K Differential Expression of HNRNPL in Response to NC, HG, and MM Conditions Analyzed by qPCR.

Supplementary Figure4

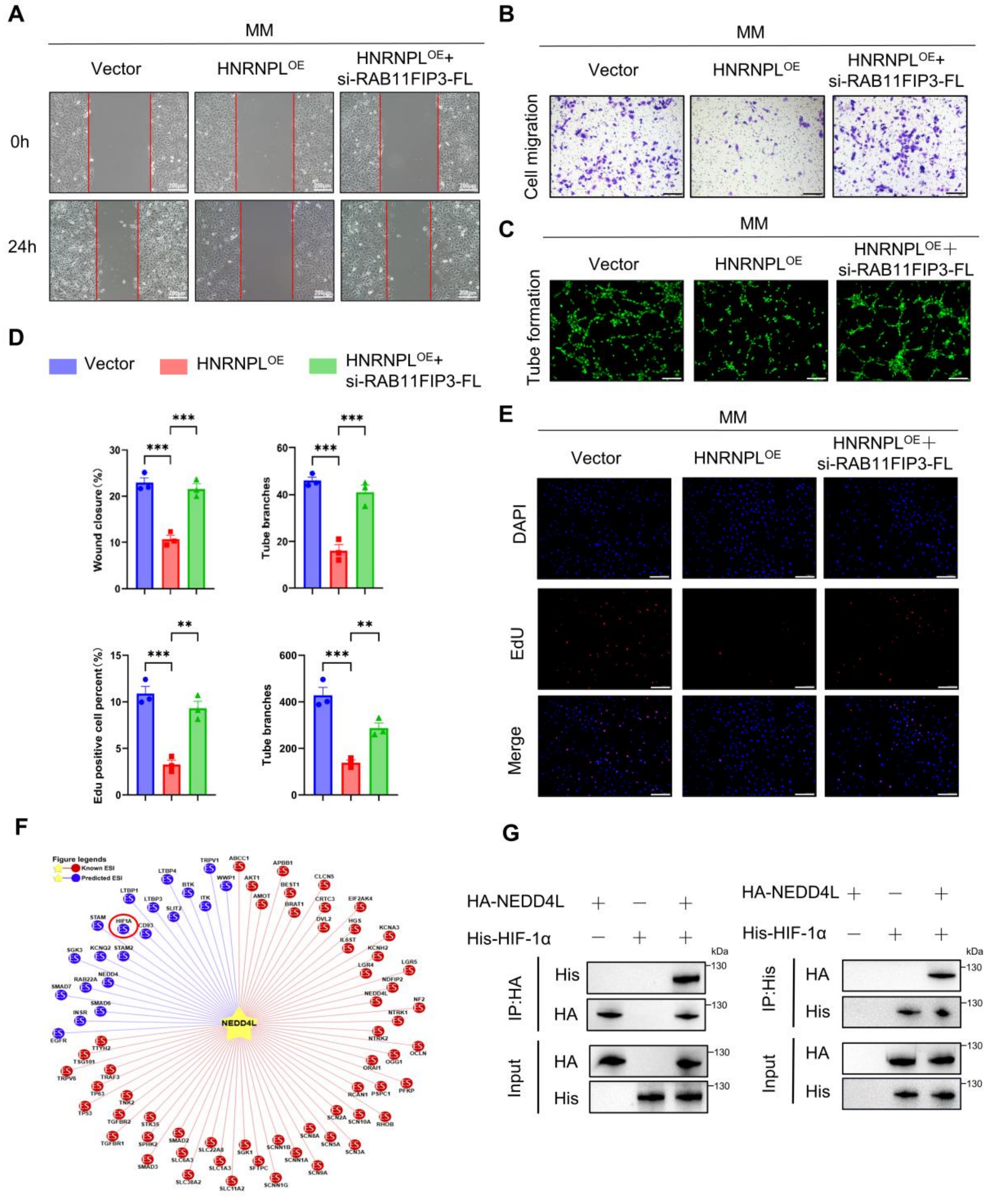

Supplementary Fig4. Functional assays and analysis of RAB11FIP3-FL interaction with NEDD4L and its effect on HUVECs behavior. A Wound healing assay of HUVECs transfected with vector, HNRNPL OE and HNRNPL OE +si-RAB11FIP3-FL. B, C transwell migration assay (B) and Angiogenesis assay (C) of HUVECs with indicated treatments. P values were calculated by two-sided Student’s t test. D Quantitative analysis of corresponding graphs A, B, C, and E. E EdU incorporation assay of HUVECs

with indicated treatments. F Predicted HIF-1α that may combine with NEDD4L through online website.

G Western blot analysis showing the interaction between RAB11FIP3-FL and NEDD4L.

Supplementary Table 1. Antibodies Used in this Study

Antibodies	SOURCE	IDENTIFIER
HNRNPL	Santa Cruz	Cat#Sc-32317
Anti-RAB11FIP3	Proteintech	Cat#25843-1-AP
Anti- NEDD4L	Proteintech	Cat#13690-1-AP
Anti-HIF-1α	Proteintech	Cat#20960-1-AP
Anti-VEGFA	Proteintech	Cat#19003-1-AP
Anti-VHL	Proteintech	Cat#24756-1-AP
Anti-Ubiquitin	Proteintech	Cat#10201-2-AP
Anti-Flag	Proteintech	Cat#66008-4-Ig
Anti-β-actin	Proteintech	Cat#66009-1-Ig
Anti-rabbit-IgG-HRP	Proteintech	Cat#SA00001-2
Anti-mouse -IgG-HRP	Proteintech	Cat#SA00001-1
Anti-HA	Proteintech	Cat#51064-2-AP
Anti-His	Biodragon	Cat#B1004

Supplementary Table 2. Primers Sequence

Gene Name	Sequence
GAPDH	5′-ATCACCATCTTCCAGGAGCGA-3′ (forward)
	5′-CCTTCTCCATGGTGGTGAAGAC-3′(reverse)
ACTB	5′-GATGCGTTGTTACAGGAAGTCC-3′ (forward)
	5′-GGCACGAAGGCTCATCATTCA-3′ (reverse)
RAB11FIP3	5′-AGCACTTTGAGGACTACGGT-3′(forward)
	5′-CAGCAATGTCCTCCTCTGG-3′(reverse)
RAB11FIP3-FL	5′-GCCAGTCTGTCATCACGGT-3′(forward)
	5′-CGCTTTGTCGGACTATTCAT-3′(reverse)
RAB11FIP3-Δ6	5′-CAGTGAGGCGGAGCTGTCCC-3′(forward)
	5′-GTACCTTGCCACCTTCTTGC-3′(reverse)
TMEM234	5′-ACCTGATGCCCTTTCTCCTC-3′(forward)
	5′-ATTCCTATCACGGTGAGCAC-3′(reverse)
TP53	5′-CAACAACACCAGCTCCTCTC-3′(forward)
	5′-CAGCTCTCGGAACATCTCGA-3′(reverse)
SNX21	5′-AAGAAGTCCCGGAACACCTT-3′(forward)
	5′-AGTAACGGCGAGAGATCTGG-3′(reverse)
TMEM40	5′-GAGACTTCAGCATCCTCCTC-3′(forward)
	5′-GAGTCCAGCCTTCCCATCTT-3′(reverse)
MORF4L2	5′-GGATTCTCGTTTCAGGTCACC-3′(forward)
	5′-AGAAGGGTGAAGTGGGAGAT-3′(reverse)

ESRP2	5´-TTGCAGCAAGGCTGATGTG-3´(forward)
	5´-GTTGAGGCAGAGTGCTACACC-3´(reverse)
HNRNPL	5´-TACGCAGCCGACAACCAAATA-3´(forward)
	5´-CTCCGGGAGTCATCCGAGT-3´(reverse)
HNRNPH1	5´-GTCCAAATAGTCCTGACACGG-3´(forward)
	5´-TCCACCGGCAATGTTATCCC-3´(reverse)
RAB11FIP3 Pre-mRNA	
Primer1	5´-ACUGCGGUCUAAAGGCAAGA-3´(reverse)
	5´-AGAAGCAUCCGUUCAGGAGA-3´(reverse)
Primer2	5´-AAAGCUGCACAGCAUCCUCA-3´(forward)
	5´-CUUUCUGCCCGCCUGAGU-3´(reverse)
Primer3	5´-GGGAGGAAGUGAUCGGUCUU-3´(forward)
	5´-CACAGUGAGCUCCCUCACAC-3´(reverse)
Primer4	5´-GAUUCCAAAGAUCCGCUAACC-3´(forward)
	5´-AGAGACUUGCCCUCCCACUC-3´(reverse)
Primer5	5´-AAAAUUAGCCAGGCAUGGUG-3´(forward)
	5´-CAGGACACACAACUGCCAGU-3´(reverse)
HIF1A	5´- CAGUCGACACAGCCUGGAUA-3´(forward)
	5´- CCACCTCTTTTGGCAAGCAT-3´(reverse)

Supplementary Table 3. siRNA and ASO Sequence

siRNA	Antisense (5´-3´)
si-RAB11FIP3-FL#1	AUUUGUUUGUGGCACUGGCTT
si-RAB11FIP3-FL#2	AUUUCAAGAUCACUCAGGCTT
si-RAB11FIP3-Δ6	UGUCGGACUGGGCGUGAGGTT
si-HNRNPL#1	UCUUCAAACUCCACCAGUGTT
si-HNRNPL#2	UGGUGAUCGAAUAAAUGGGTT
si-HNRNPL#3	UCGAUCUUCAGAGUGCAACTT
si-HNRNPH1#1	UGAAACGAAUACCUUGAGCTT
si-HNRNPH1#2	AUGGCCAUAAGCUUUCGUGTT
si-HNRNPH1#3	UAGCUCUGUAAGGUAAUCCTT
si-ESRP2#1	AUACACCUCAAUAUAGCGGTT
si-ESRP2#2	UAUCGCUUACCCAGCAUGCTT
si-ESRP2#3	UAUGGCAACGCUGAGCAGCTT
si-VHL#1	AUUGCAGAAGAUGACCUGGTT
si-VHL#2	UUGAUGUGCAAUGCGCUCCTT
si-VHL#3	UUCUCAGGCUUGACUAGGCTT
si-NEDD4L#1	UAAAUGUAUAGGGUCGCUCTT
si-NEDD4L#2	AAGCCUUCUGCUUAGUUCCTT
si-NEDD4L#3	AAGUCAAGGCGAUUAAAGCTT
ASO-RAB11FIP3-FL#1	GAUUUGTTTGTGGCACUGGC
ASO-RAB11FIP3-FL#2	CAUUUCAAGATCACTCAGGC

ASO-RAB11FIP3-Δ6	UUGUCGGACTGGGCGUGAGG

Supplementary Table 4. Summary of Sources and Catalog Numbers of experimental Reagents

PRODUCTS	SOURCE	CATALOG
DMEM	KeyGen Biotech, Jiangsu, China	KGM12800/KGL1206
fetal bovine serum	Clark Bioscience, Houston, USA	FB15015
Cell-Light EdU Apollo567 In Vitro Ki	RiboBio, Guangzhou, China	C10310-1
streptozotocin	Sigma, Missouri, USA	S0130
TBS Powder	MCE, New Jersey, USA	HY-K1026
KOD-plus-Neo	Toyobo, Osaka, Japan	KOD-401
Hieff transTM Liposomal transfection Reagent	Yeasen,Shanghai, China	40802ES03
Gel Red nucleic acid dye	VICMED, Jiangsu, China	VN302-500
Amersham Protran Premium 0.2 NC	Cytiva, Massachusetts, USA	10600001
DNA Marker I	TIANGEN, Beijing, China	MD101-01
CELLSAVING	NCM, Jiangsu, China	C40050/C40100
Basement Membrane Matrix HC	MCE, New Jersey, USA	HY-K6008
FuturePAGETM 4-20% 12 Wells	ACE, Jiangsu, China	ET12420GEL
PageRuler™	Thermo, Massachusetts, USA	26616
